# Integrated malaria service delivery and its determinants among pregnant women in Ethiopia: Multi level analysis of 2021/22 National Service Provision Assessment Survey

**DOI:** 10.1371/journal.pone.0346831

**Published:** 2026-04-15

**Authors:** Kassawmar Angaw Bogale, Kassahun Alemu, Kindie Fentahun Muchie, Mulusew Andualem Asemahagn, Hailemariam Awoke Engedaw, Muluken Azage Yenesew

**Affiliations:** 1 Department of Epidemiology and Biostatistics, School of Public Health, College of Medicine and Health Sciences, Bahir Dar University, Bahir Dar, Ethiopia; 2 Department of Epidemiology and Biostatistics, Institute of Public Health, College of Medicine and Health Sciences, University of Gondar, Gondar, Ethiopia; 3 Department for Infectious Disease and Tropical Medicine, University Hospital Heidelberg, Heidelberg, Germany; 4 Department of internal medicine, School of Medicine, College of Medicine and Health Sciences, Bahir Dar University, Bahir Dar, Ethiopia; 5 Department of Environmental Health, School of Public Health, College of Medicine and Health Sciences, Bahir Dar University, Bahir Dar, Ethiopia; 6 Department of Health system and health Economics, School of Public Health, College of Medicine and Health Sciences, Bahir Dar University, Bahir Dar, Ethiopia; Freelance Consultant, Myanmar, MYANMAR

## Abstract

**Background:**

Malaria in pregnancy poses significant health risks to mothers and their, fetuses, and newborns children in Tropical and subtropical countries including Ethiopia. The delivery of integrating essential malaria services into routine antenatal care is crucial for effective prevention and control. However, evidence on the extent and determinants of this integration in Ethiopia remains limited.

**Objective:**

This study aimed to assess the delivery of integrated malaria services during ANC visit and its determinants among pregnant women in Ethiopia.

**Methods:**

We conducted a secondary analysis of the Ethiopia Service Provision Assessment Survey 2021/22, a nationally representative cross‑sectional study. The final sample included 4273 pregnant women nested across 662 health facilities. Factors were identified based on the WHO Malaria in Pregnancy framework. Multilevel logistic regression models were applied to identify significant factors influencing integrated service uptake.

**Result:**

Only 7.9% of pregnant women attended ANC visits where all components of integrated malaria services were delivered concurrently, with substantial regional disparities. At the client level, women with two previous pregnancies (AOR = 1.67, 95% CI: 1.06–2.62), attending three or more ANC visits (AOR = 1.58, 95% CI: 1.04–2.40) and client who received an Insecticide-Treated Net during ANC (AOR = 2.81, 95% CI: 1.29–6.12) were more likely to attend ANC visits in which integrated malaria services were delivered. Furthermore, clients attending facilities with malaria-trained providers were more likely to receive integrated malaria services during ANC than those attending facilities without such training (AOR = 4.24, 95% CI: 1.80–10.00). Rural facility attendance was also positively associated with integrated malaria service delivery compared with urban facility attendance (AOR = 2.73, 95% CI: 1.04–7.19).

**Conclusion:**

Integrated malaria service delivery during ANC remains unacceptably low in Ethiopia, constrained by regional disparities and multilevel factors. Strengthen continuity of ANC follow up, updating policy on ITN distribution, strengthening providers’ capacity, and addressing geographic disparities to accelerate progress toward WHO maternal health targets.

## Background

Malaria infection during pregnancy (MiP) continues to be a significant global public health concern, particularly in sub-Saharan Africa, Southeast Asia, and parts of Latin America. Its prevalence in sub-Saharan Africa is as high as 60%, with placental malaria affecting up to 28% of cases [1,2].

MiP poses a serious threat to the health of mothers, fetuses, and newborns ranging from asymptomatic cases to severe anemia and maternal death. Physiological changes during pregnancy increase vulnerability to malaria, leading to unique challenges and severe health risks [3,4]. Clinical manifestations in pregnant women include joint pain, fever, headaches, fatigue, nausea, and anemia, with complications varying across trimesters [3,5]. Beyond maternal health, MiP contributes to unfavorable birth outcomes, including miscarriage, premature delivery, and neonatal death. It can impair placental development and function, leading to poor fetal growth, intrauterine growth restriction, and long-term infant health problems. Even in areas with low-to-moderate transmission, MiP contributes significantly to adverse outcomes including stillbirth, neonatal death, and placental insufficiency [4,6,7].

The burden of MiP is further exacerbated by socioeconomic barriers, delayed care-seeking, and limited access to integrated healthcare services delivery. Inadequate diagnosis and mismanagement of malaria cases, along with provider limitations in counseling and clinical skills, compound the challenges in effectively delivering MiP services [8]. For instance in Sub-Saharan Africa where the high malaria burden and inadequate malaria service during pregnancy reported that Low birth weight (LBW) related to malaria infection during pregnancy was accounted 35% of live births and 11% of neonatal mortality [2].

Ethiopia, home to over 132 million people, remains malaria-endemic, with 75% of its landmass conducive to transmission and an estimated 70% of pregnant women at risk [9]. The country aims to eliminate malaria among this group by 2030. While national malaria control efforts achieved substantial progress between 2000 and 2019, recent data indicate that malaria became a major public health problem for pregnant women [5,10–12].

Integration of malaria services delivery within antenatal care (ANC) is an essential strategy for improving prevention, early diagnosis, and management of malaria in pregnancy. Effective integration requires not only client engagement but also the simultaneous availability of preventive counseling, trained providers, and facility capacity to diagnose and treat malaria. According to the WHO framework, malaria service integration delivery with ANC is defined as the receipt of ANC in a setting where malaria prevention counseling, provider capability, and facility readiness were simultaneously available during the visit. [13–17].

Despite the several established advantages of malaria service integration with ANC [14–16,18–23], multilevel factors affect the integration of malaria service delivery into ANC services which leads low uptake of the service by pregnant women. From the system level factors; some countries including Ethiopia did not adopt the inclusion of IPTP and provision of ITN to their health policies [24] which affects the implementation of service integration. The availability, readiness, and actual provision of services at the facility level also affect the delivery of integrated MiP services [21,22,25–28]. The shortage of essential commodities and limited capacity of health professional to provide malarias service also affect the implementation of service integration [22,29,30]

Beyond structural and facility level influences, the effective delivery of integrated service is also shaped by client-level factors such as age, ANC follow-up adherence, pregnancy status, educational status, and awareness of malaria infection and prevention strategies [10,29,31–33].

While the WHO strongly recommends the delivery of integrating malaria services into ANC to improve prevention and treatment services, and despite malaria remaining endemic in several Ethiopian regions, evidence on the extent and the specific determinants influencing the delivery of integrated malaria service with ANC service remains limited. This study, therefore, uses nationally representative data from the 2021/2022 SPA survey to assess the level of integrated malaria services delivery with ANC and to identify the client and facility-level factors associated with such delivery of integration.

## Methods

### Data source and setting

Data were derived from the SPA Survey conducted in 2021/22 in Ethiopia. The SPA survey is a nationally representative, facility-based survey conducted by the Ethiopian Public Health Institute (EPHI) and Inner City Fund (ICF) [34]. The survey employed stratified multistage sampling to collect data from health facilities, health workers, and clients, designed to provide information on the availability and quality of health services at various levels of the healthcare system. All clients (pregnant women) visit the sampled health facilities during data collection were interviewed. The study was conducted across Ethiopia’s nine regions and two city administrations.

Data were collected through structured questionnaires administered to health facility representatives, service providers, and exit interviews with pregnant women.

### Study population and sampling

The study population included pregnant women receiving ANC care services, along with their healthcare providers and the facilities where these services were rendered. The SPA Survey employed a multi-stage cluster sampling design to select a nationally representative sample of health facilities [34]. The survey selected a stratified random sample of 1,407 health facilities, selected with probability systematic sampling. However, data was successfully collected from 1156 facilities; the remaining were permanently closed, not yet operational, under security issues, unreachable, or duplicates of another facility in the sample.

**Fig 1:**
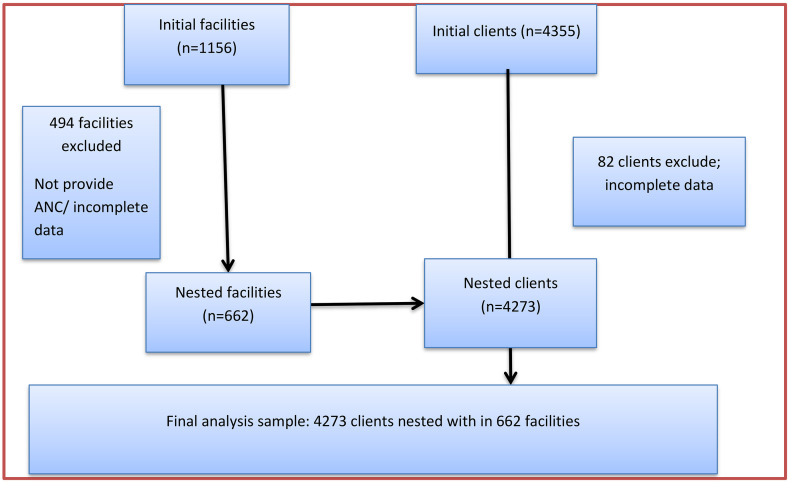
Flow diagram of the sampling structure and dataset composition for the analysis of integrated malaria service delivery among pregnant women attending ANC in Ethiopia, 2021/22.

From a total of 1156 initial facilities**,** 662 facilities were ultimately nested in the analysis. The other 494 health facilities were excluded because of they did not provide ANC services, and thus no data were collected on ANC or malaria service integration from these facilities. The number of eligible clients identified and present for ANC services was 4355**.** From this initial client pool, 4273 clients were nested within 662 facilities. The final analysis, therefore, was based on 4273 clients, nested within in 662 facilities (Fig 1).

### Variables and measurements

Integrated malaria service delivery during antenatal care (ANC) was assessed using data from the 2021–22 Ethiopia Service Provision Assessment (SPA) survey. The SPA employs standardized instruments developed by the DHS Program and collects information at three complementary levels: client exit interviews, health care provider interviews, and health facility inventories. Consistent with the WHO framework for integrated malaria services delivery for pregnant women, integration was operationalized by linking these three levels to capture whether pregnant women received coordinated malaria-related services during ANC.

#### Integrated malaria service delivery during ANC.

The concurrent presence of three malaria service components during an ANC encounter: (1) malaria prevention counseling on ITN use reported by the client, (2) provider capacity to manage malaria cases according to national guidelines, and (3) facility readiness for malaria diagnosis and treatment. These components reflect process and structural indicators of integrated service delivery derived from the client interview, provider interview, and facility inventory modules of the SPA survey.

#### Indicator 1: Malaria prevention counseling (client-level).

Malaria prevention counseling was measured using the client exit interview dataset. Pregnant women attending ANC were asked whether they received counseling on the use of insecticide-treated nets (ITNs) for malaria prevention. This variable was coded as:

- **Yes (1):** Client reported receiving counseling on ITN use
- **No (0):** Client did not receive counseling on ITN use

ITN counseling was selected as the preventive component because it is the standard malaria prevention intervention delivered within ANC services in Ethiopia, whereas ITN receipt typically occurs through community or household-level distribution campaigns. Intermittent preventive treatment in pregnancy (IPTp) is not part of Ethiopia’s national malaria policy and was therefore not included.

#### Indicator 2: Provider capacity for malaria case management (provider-level).

Provider capacity to deliver malaria case management was assessed using the health care provider interview dataset. Providers were classified as having malaria case management capacity if they reported routinely providing malaria diagnosis and treatment services as part of their clinical responsibilities.

Providers meeting these criteria were coded as:

- **Adequate capacity (1)**
- **Inadequate capacity (0)**

#### Indicator 3: Facility readiness for malaria diagnosis and treatment (facility-level).

Facility readiness was assessed using the facility inventory dataset. Facilities were classified as malaria-ready if they had:

- At least one functional malaria diagnostic method available on the day of assessment (rapid diagnostic test and/or microscopy), and
- At least one first-line antimalarial medication in stock consistent with national treatment guidelines.

Facilities meeting both criteria were coded as:

- **Ready (1)**
- **Not ready (0)**

This indicator captures the structural capacity required to support integrated malaria service delivery during ANC.

#### Construction of the integrated malaria service delivery variable.

The three indicators were combined to generate a composite measure of integrated malaria service delivery, reflecting whether malaria prevention, diagnosis, and treatment services were delivered in a coordinated manner during ANC. Integration status was classified into four mutually exclusive categories:

- **Fully integrated:** All three components present
- **Partially integrated:** Any two of the three components present
- **Limited integrated:** Only one component present
- **Not integrated:** None of the components present

For regression analyses, the outcome variable was dichotomized as follows:

Integrated service delivery (1): all three service components were present.

No integrated service delivery (0): at least one of the three service components was not present.

This approach aligns with WHO guidance on integrated service delivery and reflects meaningful thresholds for coordinated malaria care during pregnancy.

#### Study covariates.

Covariates were selected *a priori* based on the WHO malaria in pregnancy service integration framework, existing literature, and data availability in the 2021/22 Ethiopia Service Provision Assessment. Variables were grouped into client-level and facility-level factors to reflect the hierarchical structure of the data.

#### Client-level covariates.

Client-level variables were extracted from the ANC client exit interview dataset and included:

- Maternal age group (15–24, 25–34, ≥ 35 years)
- Educational status (no formal education, primary(grades (1–8), secondary (9–12) or higher (diploma and above)
- Marital status (married vs. not married)
- Pregnancy status (gravida) (primigravida vs. multigravida)
- Number of ANC visits (1, 2, ≥ 3 visits)
- Receipt of insecticide-treated net (ITN) during ANC (yes/no)
- Knowledge of fever as a danger sign in pregnancy (yes/no)
- Payment for ANC services (yes/no)

#### Facility-level covariates.

Facility-level variables were obtained from the SPA facility inventory and provider interview datasets and included:

- Facility location (urban/rural)
- Facility ownership (public vs. private/NGO)
- Facility category (hospital vs. health center)
- Availability of malaria diagnostics (microscopy or rapid diagnostic test)
- Availability of first-line antimalarial drugs in line with national guidelines
- Facility malaria training: defined as the presence of at least one ANC service provider at the facility who reported receiving formal training on malaria diagnosis and treatment within the past 24 months (yes/no)
- Supervision received: defined as whether the facility or ANC provider reported receiving any external supervisory visit related to maternal or malaria services within the last six months (yes/no)

All facility- and provider-level variables were linked to clients through the facility identifier and treated as higher-level covariates in the multilevel analysis

#### Operational definition.

**Integrated malaria Service Delivery:**

Delivery of coordinated malaria- services during ANC, defined by the concurrent presence of malaria prevention counseling, provider capacity for malaria case management, and facility readiness for malaria diagnosis and treatment.

**Malaria burden regions** were operationally defined as a categorical variable with three levels: “High,” “Moderate,” and “Low.” This classification was based on the endemicity and malaria transmission intensity observed across Ethiopia’s administrative regions, aligned with the national malaria stratification [35].

- **High Burden Region:** Regions characterized by high prevalence/incidence and higher caseloads. These include Gambela, Benishangul-Gumuz, Amhara, Oromia, and Southern Nations, Nationalities, and Peoples’ Region (SNNPR) regional states.
- **Moderate Burden Region:** Regions with moderate case numbers. These include Somalia, Sidama, and Afar regional states.
- **Low Burden Region:** Regions with sporadic or minimal malaria transmission. These include Addis Ababa, Dire Dawa city administrations, and Harari regional state.

### Data management and analysis

Missing data management was employed using different techniques. Variables with minimal missingness (<1) (e.g., diagnostic availability, supervision) were conservatively recoded, while those with moderate missingness (> 1) (e.g., guideline availability, training) were addressed using Multiple Imputation by Chained Equations (MICE) with logistic regression models [36]. The outcome variable was recomputed in each imputed dataset prior to modeling.

Health care provider data were first linked to the respective facilities and then aggregated to represent facility-level characteristics. The presence of multicollinearity among independent variables was assessed using Generalized Variance Inflation Factors (GVIF). All GVIF values were well below the common threshold of 5, indicating no significant multicollinearity issues.

Descriptive statistics were used to summarize the characteristics of the study population, with categorical variables presented as frequencies and percentages. A forest plot was used to display the regional distribution of malaria service integration among antenatal care (ANC) users. In addition, bar charts were used to illustrate the status of malaria service integration across regions.

Multilevel logistic regression analysis was employed to identify factors associated with the receipt of integrated malaria services delivery, accounting for the hierarchical structure of the data where clients were nested within facility. The analysis was conducted using statistical software R (version 4.3) with the *lme4* package for mixed-effects models.

We used the incremental value of adding different levels of predictors, three multilevel logistic regression models were compared: a null model (intercept only), a client-level model (model I), and a full model (combining client and facility-level predictors). The comparison of the multilevel logistic regression models demonstrated that the facility-level clustering accounted for a substantial proportion of the total variation in integrated malaria services delivery, as reflected by the high intra class correlation coefficient (ICC) of 0.934 in the null model. When client-level and facility-level predictors were sequentially added, the conditional R^2^ remained high (0.907 in the final model), indicating that the random effects at the facility level continued to explain most of the variability. However, the marginal R^2^ increased from 0.000 in the null model to 0.022 in the full model, showing that the inclusion of fixed predictors modestly improved the model’s ability to explain variation in integration status. The root mean square error (RMSE) decreased slightly from 0.191 to 0.190, suggesting improved predictive performance. Model selection criteria further supported the final model; it had the lowest AIC indicating a superior balance of goodness of fit.

Adjusted Odds Ratios (AORs) with their corresponding 95% Confidence Intervals (CIs) and p-values were reported for fixed effects in the final model. We conducted bivariate analyses, and variables with a p-value ≤ 0.2 were retained for final modeling. A p-value of less than 0.05 was considered statistically significant.

### Ethical considerations

The 2022 Ethiopia SPA Survey received ethical clearance from the EPHI and the Institutional Review Board of ICF International. For this secondary analysis, anonyms and publicly available data were used, and no additional ethical approval and consent was required [37].

## Results

### Socio-demographic and client-level characteristics

The study analyzed data from 4,273 pregnant women who received ANC services in Ethiopia, based on the 2021/2022 Ethiopia SPA survey. The analysis of client-level characteristics provides insights into the socio-demographic profile and service utilization patterns of the participants.

The mean age of the client was 25.69 years (SD = 4.99), with the largest proportion—1,618 women (37.86%)—falling within the 25–29 years age group. Regarding educational status, 1,475 participants (34.52%) had attained secondary education. The majority of respondents were from urban areas 2,891 (67.65%), and most were multigravidas, accounting for 2,971 women (69.53%).

In terms of ANC utilization, nearly half 2,033 (47.58%) of the women had attended only one ANC visit. Alarmingly, a significant majority, 3,847 women (90%) were unaware that fever is a danger sign during pregnancy. Additionally, most participants 3,415 (79.89%) got ANC service free of payment.

Concerning malaria prevention, the findings reveal substantial service gaps. Only 123 women (2.88%) reported receiving insecticide-treated nets (ITNs) at the health facility, and just only 859 women (20%) received counseling on ITN use, a key intervention for malaria prevention during pregnancy (Table 1).

**Table 1:** Socio-demographic and client-level characteristics of pregnant women attending ANC in Ethiopia, 2021/22 National Service Provision Assessment (N = 4,273).

Characteristic	Category	Frequency (N)	Percentage (%)
Maternal Age Group	<25 years	1,618	37.86
	25–29 years	1,627	38.08
	>30 years	1,028	24.06
Education Level	No education	911	21.32
	Primary (grade 1–8)	1,361	31.85
	Secondary (grade 9–12)	1,475	34.52
	Higher (college and above)	526	12.31
Gravidity	1	1,302	30.47
	2	1194	27.94
	3 or more	1777	41.59
ANC Visits	1	2,033	47.58
	2	1,001	23.43
	3	630	14.74
	4 or more	609	14.25
Paid for ANC	No	3,415	79.92
	Yes	858	20.08
Knows Fever as Danger Sign	No	3,847	90.00
	Yes	426	10.00
Received ITN	No	4,150	97.12
	Yes	123	2.88
Counseled ITN use	No	3,414	79.90
	Yes	859	20.10

### The distribution of facilities and clients in Ethiopia

The distribution of clients were varied by regions, For example, the Afar region had the smallest sample size with 85 clients, while the Oromia region represented the largest portion of the study population with 1,176 clients (Table 2).

**Table 2:** Distribution of health facilities and ANC clients across regions in Ethiopia, 2021/22 National Service Provision Assessment Survey.

Region	Facilities Included	Total Clients
Afar	24	85
Amhara	108	683
Oromiya	171	1376
Somali	41	220
Benishangul	21	115
SNNP	106	781
Gambella	35	115
Harari	17	80
Addis Ababa	52	379
Dire Dawa	27	126
Sidama	60	313
**Total**	**662**	**4273**

Facility-level characteristics provide critical insights into the healthcare system environment that pregnant women attended ANC visits where integrated malaria services were delivered. The vast majority of clients 4,157 (97.29%) received services from facilities that offered malaria case management services, including diagnosis and treatment. Additionally, 2,980 (69.74%) of the women received care at facilities equipped with malaria diagnostics (microscope or RDT). Hospitals accounted for the largest share of service locations 3,009 (70.42%). In terms of ownership, governmental facilities dominated the service landscape, with 3,774 (88.32%) of women attending these institutions, while private or NGO facilities served a smaller portion (499; 11.68%).

Geographically, majority services were delivered in urban areas, where 2,891 (67.65%) of clients were seen. Notably, 3,070 women (71.85%) were seen at facilities located in high malaria burden regions (Table 3).

**Table 3:** Facility-level characteristics related to integrated malaria service delivery during ANC among pregnant women in Ethiopia, 2021/22 National Service Provision Assessment Survey (N = 4,273).

Characteristic	Category	Frequency (N)	Percentage (%)
Malaria tx Services avai	No	116	2.71
	Yes	4,157	97.29
Malaria Test Lab avai	No	1,293	30.26
	Yes	2,980	69.74
Facility Category	Hospital	3,009	70.42
	Other Health Facility	1,264	29.58
Ownership	Governmental	3,774	88.32
	Private/NGO	499	11.68
Location	Urban	2,891	67.65
	Rural	1,382	32.34
Facility in Malaria Burden Region	Low	585	13.68
	Moderate	618	14.47
	High	3,070	71.85

Facility malaria tx services” refers to the availability of malaria treatment services, while “malaria test lab availability” refers to the presence of laboratory capacity for malaria testing.

### Classification and distribution of malaria service delivery

**Fig 2:**
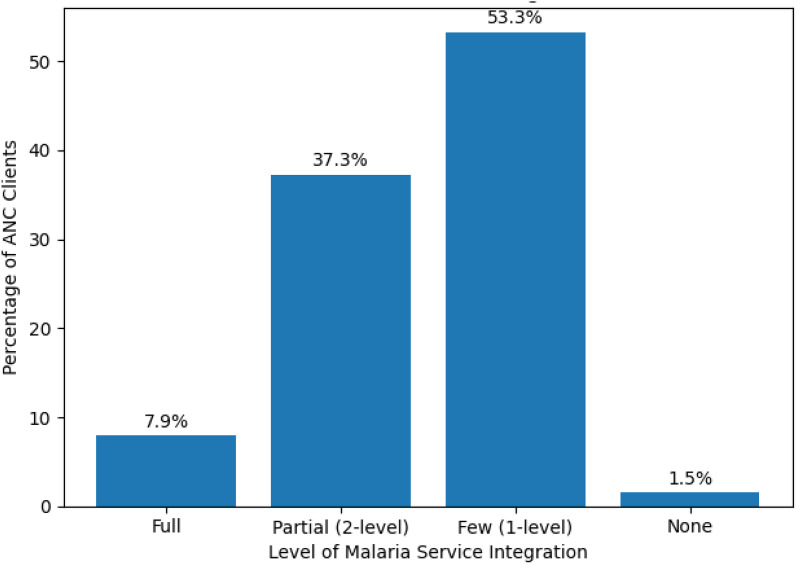
Classification and distribution of integrated malaria service delivery during antenatal care among pregnant women in Ethiopia, 2021/22 National Service Provision Assessment Survey.

As shown in Fig 2, the majority of pregnant women (53.3%) received ANC services in facilities classified as few integrated, where only one malaria service component was available.

An additional 37.3% of ANC clients accessed partially integrated. Only 7.9% of pregnant women attended ANC visits where integrated malaria services were delivered.Conversely, 1.5% of clients received ANC services in facilities with no malaria service integration (Fig 2).

### Integrated malaria ServiceDelivery and disparities across regions in Ethiopia

Nationally, only 7.9% (95% CI: 7.1%–8.7%) of pregnant women received ANC care visits where fully integrated malaria services were delivered.

**Fig 3:**
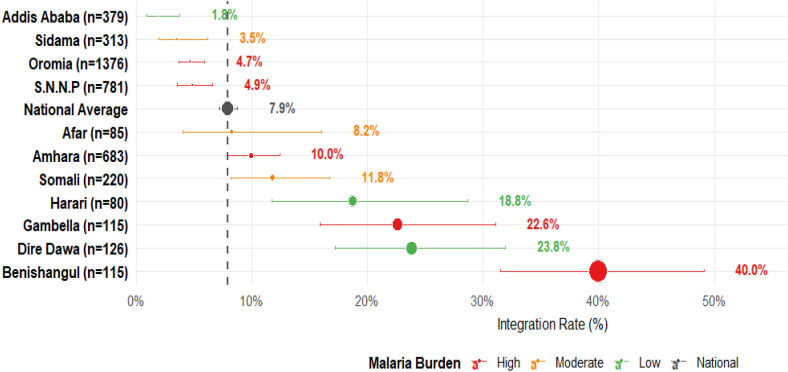
Forest plot showing the proportion of integrated malaria service delivery during ANC by region in Ethiopia, 2021/22 National Service Provision Assessment Survey.

Considerable regional disparities were observed in the delivery of integrated malaria services during ANC across Ethiopia. As presented in Fig 3, the proportion of clients receiving integrated malaria services exceeded the national average in several regions. The highest coverage was recorded in Benishangul Gumuz at 40.0% (95% CI: 31.5%–49.1%), followed by Dire Dawa at 23.8**%** (95% CI: 17.2%–32.0%), Gambella **at** 22.6% (95% CI: 15.9%–31.1%), and Harari **at** 18.8**%** (95% CI: 11.7%–28.7%).

Conversely, pregnant women who visited facilities in four regions exhibit integrated malaria service Delivery proportions considerably lower than the national average. Client visit health facilities in Addis Ababa registered the lowest integration proportions at 1.8% [95% CI: 0.9%, 3.8%], followed by Sidama at 3.5% [95% CI: 2.0%, 6.2%], Oromia at 4.7% [95% CI: 3.7%, 5.9%], and S.N.N.P. at 4.9% [95% CI: 3.6%, 6.6%]**.** The disparities highlight the statistical significance of these regional differences compared to the national average malaria integration (Fig 3).

### Multilevel logistic regression analysis

The multilevel logistic regression analysis model examined factors associated with the delivery of integrated malaria services among ANC clients, accounting for both client-level and facility-level influences. In model, where client- and facility-level factors were simultaneously included, five factors were found to be significantly associated with the integration of malaria services (p < 0.05).

At the client level, pregnancy status (gravida), number of ANC visits, and receipt of ITNs were significant predictors. Women with two previous pregnancies were 1.67 times more likely than primigravida women to attend visits in which integrated malaria services were delivered (AOR = 1.67, 95% CI: 1.06–2.62).

Similarly, attendance at three or more ANC visits was associated with higher odds of receiving care during visits in which fully integrated malaria services were delivered, compared with attendance at only one ANC visit (AOR = 1.58; 95% CI: 1.04–2.40). Receipt of an ITN during ANC was also positively associated with integrated malaria service delivery (AOR = 2.81; 95% CI: 1.29–6.12).

At the facility level, providers training on malaria and facility location were significantly associated with service integration delivery.

Clients attending facilities with malaria-trained providers had significantly higher odds of receiving integrated malaria services during ANC than those attending facilities without trained providers (AOR = 4.24; 95% CI: 1.80–10.00). Likewise, clients receiving ANC in rural facilities were more likely to experience integrated malaria service delivery than those in urban facilities (AOR = 2.73; 95% CI: 1.04–7.19). In contrast, knowledge of fever as a danger sign, facility supervision, facility category, and ownership were not significantly associated with integrated malaria service delivery.

(Table 4).

**Table 4:** Multilevel determinants of integrated malaria service delivery during ANC among pregnant women in Ethiopia, 2021/22 National Service Provision Assessment Survey.

Variables	Category	Model 1 AOR (95% CI)	Final Model AOR (95% CI)
Gravida	1 (Ref)	1	1
	2	**1.61 (1.05–2.45)**	**1.67 (1.06–2.62)**
	3 or more	1.25 (0.83–1.90)	1.33 (0.86–2.05)
ANC visits	1 (Ref)	1	1
	2	1.15 (0.78–1.71)	1.30 (0.84–1.99)
	3 or more	1.29 (0.89–1.88)	**1.58 (1.04–2.40)**
Knows fever danger sign	No (Ref)	1	1
	Yes	1.11 (0.70–1.76)	1.16 (0.65–2.07)
Received ITN	No (Ref)		
	Yes	**2.25 (1.22–4.15)**	**2.81 (1.29–6.12)**
Supervision received	No (Ref)	1	1
	Yes	—	1.44 (0.82–2.50)
Facility training malaria	No (Ref)	1	1
	Yes	—	**4.24 (1.80–10.0)**
Facility category (Other)	Hospital	1	1
	Others	—	1.99 (0.77–5.12)
Ownership of facility	Government		
	Private/NGO	—	1.81 (0.53–6.16)
Location of facility	Urban	1	1
	Rural	—	**2.73 (1.04–7.19)**

## Discussion

This study utilized a nationally representative dataset from the 2021/22 Ethiopia SPA survey to assess the level of integration of malaria services delivery during ANC and to identify the multifaceted client and facility-level factors influencing this delivery.

The delivery of integrated malaria services into ANC remains critically limited in Ethiopia. This low figure underscores missed opportunities for delivering comprehensive malaria interventions at a critical point of maternal care. This indicates a substantial gap in the country’s efforts to safeguard pregnant women from malaria and achieve its ambitious 2030 malaria elimination goals, aligning with the observed re-emergence of malaria as a top health concern despite previous progress [38].

The most common pattern observed was limited integration, accounting for 53.3% of all ANC visits. In these cases, although facilities were capable of providing malaria diagnosis and treatment, there was a lack of provider-level engagement or client counseling on malaria prevention. This finding indicates that infrastructure alone is insufficient to guarantee service delivery, and highlights the need to strengthen the human resource and communication components of ANC visits. The findings is consistent with the analysis of service integration delivery in SSA [39].

These findings reflect systemic challenges in aligning policy, capacity, and practice. Despite WHO recommendations to integrate malaria services—particularly IPTp, case management, and promotion and use of ITN —within ANC [17], the Ethiopian context lacks full policy adoption, particularly the absence of IPTp-SP and ITN provision at ANC in national guidelines [24]. Furthermore, the study revealed significant regional disparities in delivery of integration services across Ethiopia.

The delivery of integrated malaria services during ANC visits was markedly higher in some regions, indicating significant regional variation relative to the national average. The heterogeneity observed in malaria service integration across regions may stem from various factors. For regions with historically low malaria burden, such as Addis Ababa, the low service integration might be explained by an assumed minimal risk, leading to reduced programmatic focus or awareness regarding malaria in pregnancy. However, the critically low integration delivery was observed in regions like Oromia and S.N.N.P are particularly concerning given that these are recognized as high malaria burden regions. This paradox suggests that despite a high epidemiological need, the health systems in these areas may be overwhelmed, prioritizing acute case management over the integration of preventive and diagnostic services into routine ANC. This could also indicate a lack of specific resource allocation or targeted strategies for MiP within these high-burden contexts. While the delivery of integrated services in regions like Amhara showed a slightly higher (10%) than the national average, it remains critically low when compared to WHO recommendations, which advocate for every pregnant woman to receive comprehensive integrated services during ANC [40].

This overall scenario strongly indicates that the Ethiopian health system might currently place greater emphasis on general population malaria prevention and treatment strategies, with a notable absence of specific, robust guidelines and programs designed to support the seamless integration of malaria services into ANC. This is further supported by qualitative findings from Ethiopia, which highlight the absence of malaria-specific interventions tailored for pregnant women, leaving them vulnerable to inadequate protection and care [29].

The multilevel logistic regression analysis revealed important determinants of malaria service integration among pregnant women attending antenatal care (ANC).

Women with two previous pregnancies (Gravida 2) and those with three or more ANC visits were more likely to receive care during visits in which fully integrated malaria services were delivered. This could suggest that multiparous women, having navigated the healthcare system before, are more adept at accessing comprehensive care, or that providers are more likely to offer extensive services to women with established records. The findings is consistent with the previous study established, pregnant women previous service experiences are favorable contributor for service integration [41].

However, this finding contrasts with the widely accepted recommendation that primigravidae should be prioritized for malaria prevention due to their greater susceptibility to malaria infection and its complications**.** The immune-naïve status of first-time pregnant women places them at higher risk for severe disease and adverse pregnancy outcomes [42,43].

On the other side, those attending three or more ANC visits were 1.48 times more likely to receive care during visits in which fully integrated malaria services were delivered. It’s plausible that as women engage more consistently with ANC services, opportunities for integrated malaria services naturally increase due to repeated interactions with the health system. These findings align with prior finding indicating that increased contact with health facilities enhances access to health education, malaria screening, and preventive interventions [10,44]. The association may also reflect the cumulative benefit of repeat ANC exposure in promoting integrated care.

The receiving an ITN during ANC were 2.81 times more likely receiving care during visits in which fully integrated malaria services were delivered. This finding suggests the provision of an ITN may serve as an entry point of a more comprehensive, integrated approach to malaria prevention and control within ANC. It is possible that facilities prioritizing ITN distribution are also more likely to be implementing other components of integrated malaria services, such as counseling and diagnosis. This finding is in line with previous evidence showing that integrated antenatal and malaria services are positively associated with improved ITN access [45].

At the facility level, this study uncovered important disparities in the delivery of integrated malaria services for pregnant women across geographic and epidemiologic contexts.

The finding reveled that clients who visit facilities where providing malaria training were significantly more likely to received care during visits in which fully integrated malaria services were delivered. This aligns with global recommendations emphasizing the need for skilled healthcare workers to deliver quality and integrated care [27,39].

Pregnant women who got service in rural facilities were significantly more likely to received care during visits in which fully integrated malaria services were delivered compared to their urban counterparts. This finding may reflect the targeted public health strategies implemented in rural areas, where maternal and malaria-related mortality have historically been higher [46]. Additionally, rural health facilities may experience lower patient volumes, allowing for longer consultation times and more opportunities for integrated service delivery [47]. However, there are contradict findings showed the rural health facilities are limited in accessing of qualified professional, essential commodities that affect the delivery of integrated service [48].

### Strengths and limitations

This study’s strengths include its use of nationally representative data from the recent 2021/22 SPA survey, providing robust generalizability. The large sample size and the application of multilevel logistic regression adequately addressed the hierarchical nature of the data, minimizing bias from clustering effects. Furthermore, the comprehensive inclusion of client and facility-level variables allowed for a nuanced understanding of the complex determinants.

Nevertheless, certain limitations should be acknowledged. Its cross-sectional design precludes the establishment of causal relationships between the identified factors and service integration and the SPA survey may not fully align with Ethiopia seasonal variation in malaria transmission; this could have influence the malaria service integration practice. In addition, the reliance on facility-based surveys and client exit interviews may be subject to recall bias or social desirability bias. Moreover, the composite operational definition of “integrated malaria services delivery” was stringent, which might contribute to the low overall integration rate. Finally, despite the use of multilevel modeling to account for clustering of clients within facilities, residual unmeasured facility- or community-level factors may still influence the observed associations.

Further research using regionally powered surveys or routine health information system data is recommended to generate more robust subnational estimates and to better understand geographic inequities in malaria service integration with ANC services.

## Conclusion

The delivery of integrated malaria services into antenatal care in Ethiopia remains critically low, marked by significant regional disparities and multilevel determinants. Key factors influencing service integration delivery include gravida, ANC visit frequency, and the provision of ITNs at the client level. At the facility level, malaria-specific training and rural location significantly impacted service integration. Addressing these complex challenges necessitates a multi-pronged approach: (i) Policy and Service Readiness: National and regional health authorities should prioritize ensuring that all ANC facilities are equipped with malaria diagnostics, treatment, and trained providers to delivered consistent and integrated services; (ii) Capacity building: continuous in-service training on MiP, including counseling on ITN use and case management, should be integrated into routine professional development programs; (iii) Equity Service delivery: regional disparities highlights the needs of intensified support in high-burden and underserved areas through targeted resource allocation, supportive supervision, and capacity building; (iv) Client Engagement: strengthening demand side intervention through promoting early and consistent ANC attendance and revising national polices to formally integrate ITN distribution into ANC follow up; (v) Health System adaptation: successful rural facility practices should be adapted and scaled to urban and high-burden regions where integration lags and (vi) Future research: Further studies should explore underlying causal pathways to fully understand the determinants of delivery of malaria service integration for pregnant women in Ethiopia.

## References

[1] Varo R, Chaccour C, Bassat Q. Update on malaria. Med Clin (Barc). 2020;155(9):395–402. doi: 10.1016/j.medcli.2020.05.010 32620355

[2] Bakken L, Iversen PO. The impact of malaria during pregnancy on low birth weight in East-Africa: a topical review. Malar J. 2021;20(1):348. doi: 10.1186/s12936-021-03883-z 34429121 PMC8386002

[3] Kamanzi Nyirabashitsi I. Clinical manifestations and health impact of malaria in pregnant women. Appl Sci (NIJBAS). 2024;5(2).

[4] Bauserman M, Conroy AL, North K, Patterson J, Bose C, Meshnick S. An overview of malaria in pregnancy. Semin Perinatol. 2019;43(5):282–90. doi: 10.1053/j.semperi.2019.03.018 30979598 PMC7895297

[5] Almaw A, Yimer M, Alemu M, Belay H, Alebachew M, Abeje G, et al. Prevalence of clinical malaria and associated symptoms in pregnant women at Hamusit health center, Northwest Ethiopia. Heliyon. 2024;10(14):e34240. doi: 10.1016/j.heliyon.2024.e34240 39816346 PMC11734082

[6] Rogerson SJ, Desai M, Mayor A, Sicuri E, Taylor SM, van Eijk AM. Burden, pathology, and costs of malaria in pregnancy: new developments for an old problem. Lancet Infect Dis. 2018;18(4):e107–18. doi: 10.1016/S1473-3099(18)30066-5 29396010

[7] Steketee RW, Nahlen BL, Parise ME, Menendez C. The burden of malaria in pregnancy in malaria-endemic areas. Am J Trop Med Hyg. 2001;64(1-2 Suppl):28–35. doi: 10.4269/ajtmh.2001.64.28 11425175

[8] Minwuyelet A, Yewhalaw D, Siferih M, Atenafu G. Current update on malaria in pregnancy: a systematic review. Trop Dis Travel Med Vaccines. 2025;11(1):14. doi: 10.1186/s40794-025-00248-1 40399982 PMC12096600

[9] Ministry of Health. National malaria elimination strategic plan 2024/25-2026/27. Ministry of Health: Addis Abab. 2023.

[10] Kassie GA, Adella GA, Gebrekidan AY, Gebeyehu NA, Gesese MM, Abebe EC, et al. Insecticide-treated bed net utilization and associated factors among pregnant women in Ethiopia: a systematic review and meta-analysis. Malar J. 2023;22(1):223. doi: 10.1186/s12936-023-04655-7 37533029 PMC10398969

[11] Ministry of Health. Ethiopia malaria elimination-strategic plan 2021 to 2025. Addis Ababa: Ministry of Health. 2020.

[12] Sisay M, Kebede M, Muluneh AG. Correction: Prevalence of malaria and associated factors among pregnant women in East Dembia District Northwest Ethiopia. BMC Pregnancy Childbirth. 2025;25(1):178. doi: 10.1186/s12884-025-07326-4 39966832 PMC11837629

[13] Ansah EK, Moucheraud C, Arogundade L, Rangel GW. Rethinking integrated service delivery for malaria. PLOS Glob Public Health. 2022;2(6):e0000462. doi: 10.1371/journal.pgph.0000462 36962405 PMC10021790

[14] Olapeju B, Bride M, Gutman JR, Wolf K, Wabwire S, Atobrah D, et al. WHO antenatal care policy and prevention of malaria in pregnancy in sub-Saharan Africa. Malar J. 2024;23(1):218. doi: 10.1186/s12936-024-05037-3 39044194 PMC11264419

[15] Quakyi I, Tornyigah B, Houze P, Kusi KA, Coleman N, Escriou G, et al. High uptake of Intermittent Preventive Treatment of malaria in pregnancy is associated with improved birth weight among pregnant women in Ghana. Sci Rep. 2019;9(1):19034. doi: 10.1038/s41598-019-55046-5 31836735 PMC6911095

[16] Odjidja EN, Gatasi G, Duric P. Delivery of integrated infectious disease control services under the new antenatal care guidelines: a service availability and readiness assessment of health facilities in Tanzania. BMC Health Serv Res. 2019;19(1):153. doi: 10.1186/s12913-019-3990-8 30866924 PMC6417178

[17] WHO. Recommendations on antenatal care for a positive pregnancy experience: screening, diagnosis, and treatment of tuberculosis disease in pregnant women. Geneva: World Health Organization. 2023. 28079998

[18] Onyinyechi OM, Ismail S, Nashriq Mohd Nazan AI. Prevention of malaria in pregnancy through health education intervention programs on insecticide-treated nets use: a systematic review. BMC Public Health. 2024;24(1):755. doi: 10.1186/s12889-024-17650-7 38468243 PMC10929229

[19] Mkubwa B, Kagura J, Chirwa T, Ibisomi L, Kinyanjui S. Determinants of utilization of malaria preventive measures during pregnancy among women aged 15 to 49 years in Kenya: an analysis of the Malaria Indicator Survey 2020. Malar J. 2022;21(1):398. doi: 10.1186/s12936-022-04425-x 36581863 PMC9798621

[20] Ampofo GD, Osarfo J, Aberese-Ako M, Asem L, Komey MN, Mohammed W, et al. Malaria in pregnancy control and pregnancy outcomes: a decade’s overview using Ghana’s DHIMS II data. Malar J. 2022;21(1):303. doi: 10.1186/s12936-022-04331-2 36303165 PMC9615308

[21] Jerene D, Fentie G, Teka M, Girma S, Chibsa S, Teka H, et al. The role of private health facilities in the provision of malaria case management and prevention services in four zones of Oromia Regional State, Ethiopia. Int Health. 2012;4(1):70–3. doi: 10.1016/j.inhe.2011.11.001 24030883

[22] Usman R, Umar AA, Gidado S, Gobir AA, Obi IF, Ajayi I, et al. Predictors of malaria Rapid Diagnostic Tests’ utilisation among healthcare workers in Zamfara State. PLoS One. 2018;13(12):e0200856. doi: 10.1371/journal.pone.0200856 30550562 PMC6294357

[23] Bogale KAA, Muluken, Alemu K, Andualem M, Worku M. Malaria Infection during Pregnancy in Global Endemic Regions: Systematic Review and Meta-Analysis. 2024.

[24] Ministry of Health. National malaria guidelines of Ethiopia. Addis Ababa: Ministry of Health. 2022.

[25] Azizi H, Majdzadeh R, Ahmadi A, Esmaeili ED, Naghili B, Mansournia MA. Health workers readiness and practice in malaria case detection and appropriate treatment: a meta-analysis and meta-regression. Malar J. 2021;20(1):420. doi: 10.1186/s12936-021-03954-1 34689791 PMC8543935

[26] Taylor C, Linn A, Wang W, Florey L, Moussa H. Examination of malaria service utilization and service provision: An analysis of DHS and SPA data from Malawi, Senegal, and Tanzania. Malar J. 2019;18:1–13. 31358005 10.1186/s12936-019-2892-x PMC6664566

[27] Mohamoud AM, Yousif MEA, Saeed OK. Effect of In-Service Training Program on the Practice of Healthcare Workers toward Malaria Prevention and Treatment Guidelines during Pregnancy in Health Facilities in Jowhar District, Somalia. Health. 2022;14(11):1173–90. doi: 10.4236/health.2022.1411083

[28] Bajaria S, Festo C, Mrema S, Shabani J, Hertzmark E, Abdul R. Assessment of the impact of availability and readiness of malaria services on uptake of intermittent preventive treatment in pregnancy (IPTp) provided during ANC visits in Tanzania. Malar J. 2019;18(1):229. doi: 10.1186/s12936-019-2862-3 31288835 PMC6617666

[29] Bogale KA, Yenesew MA, Alemu K, Muchie KF, Asemahagn MA, Enbiale W. Facilitators and barriers of malaria prevention and treatment services to pregnant women in Ethiopia: a multi-level health system analysis, 2025. Malar J. 2025;24(1):337. doi: 10.1186/s12936-025-05555-8 41088198 PMC12522862

[30] Tesha GE, Makwaruzi S, Haws R, Mostel J, Lusasi A, Lazaro S, et al. Understanding Antenatal Care Service Quality for Malaria in Pregnancy through Supportive Supervision Data in Tanzania. Am J Trop Med Hyg. 2024;110(3_Suppl):56–65. doi: 10.4269/ajtmh.23-0399 38320309 PMC10919228

[31] Bogale KA, Asemahagn MA, Gelaye KA, Muchie KF, Engedaw HA, Azage M. Malaria service readiness and associated factors among health facilities that provide antenatal care services in Ethiopia: a cross-sectional study using generalised estimating equation analysis. BMJ Open. 2026;16(2):e109109. doi: 10.1136/bmjopen-2025-109109 41644155 PMC12878252

[32] Negasa K, Huluka TK, Yebassa MA, Waqkene T. Utilization of long-lasting insecticide-treated net and its associated factors among pregnant women in Dawo district, Southwest Shoa Zone, Oromia, Ethiopia, 2023. Front Public Health. 2024;11:1261254. 38348378 10.3389/fpubh.2023.1261254 PMC10859520

[33] Shonga AA, Nahusenay H, Tadesse M. Insecticide Treated Bed Nets (ITNs) Utilization and Associated Factors Among Pregnant Mothers in Damot Pulasa District, Southern Ethiopia. 2018. https://example.com/district-southern-ethiopia-2018

[34] Ethiopian Public Health Institute, Ministry of Health E, ICF. Ethiopia service provision assessment 2021–22 final report. Addis Ababa, Ethiopia, and Rockville, Maryland, USA: EPHI, MoH and ICF. 2023.

[35] Ministry of Health. Targeted cluster approach and strategic advocacy to curb the malaria surge in high-burden and conflict-affected areas. Addis Abeba: Ministry of Health Ethiopia. 2024.

[36] Wulff JN, Ejlskov L. Multiple imputation by chained equations in praxis: guidelines and review. Bus Res Methods. 2017;15(1):41–56.

[37] Ethiopia Public Health Institute. Service Provision Assessment in Ethiopia. Addis Ababa, Ethiopia: Ethiopia Public Health Institute. 2022.

[38] Woldesenbet D, Tegegne Y, Mussema A, Tamene E, Mohamed K, Abebe W. Can Ethiopia eliminate malaria? Malaria burden: insights from the pre-elimination era, current challenges and perspectives. Front Malar. 2025;3:1492444.

[39] Xu X, Liang D, Zhao J, Mpembeni R, Olenja J, Yam EL, et al. The readiness of malaria services and uptake of intermittent preventive treatment in pregnancy in six sub-Saharan countries. J Glob Health. 2024;14:04112. doi: 10.7189/jogh.14.04112 38939971 PMC11211972

[40] Ntirushwa D. A strategic framework for malaria prevention and control during pregnancy in the African region. WHO Regional Office for Africa. 2004.

[41] BogaleKA, Asemahagn, MuchieMAK, EngdawK, YenesewH, Muluken. Integrated malaria service uptake and its determinants among pregnant women in Ethiopia: Multi level analysis of 2021/22 National Service Provision Assessment. In: Bahir Dar University E. Bahir Dar.10.1371/journal.pone.034683141984977

[42] Moore KA, Fowkes FJI, Wiladphaingern J, Wai NS, Paw MK, Pimanpanarak M, et al. Mediation of the effect of malaria in pregnancy on stillbirth and neonatal death in an area of low transmission: observational data analysis. BMC Med. 2017;15(1):98. doi: 10.1186/s12916-017-0863-z 28486979 PMC5424335

[43] Limenih A, Gelaye W, Alemu G. Prevalence of Malaria and Associated Factors among Delivering Mothers in Northwest Ethiopia. Biomed Res Int. 2021;2021:2754407. doi: 10.1155/2021/2754407 34917681 PMC8670928

[44] Nigatu AM, Gelaye KA. Factors associated with the preference of institutional delivery after antenatal care attendance in Northwest Ethiopia. BMC Health Serv Res. 2019;19(1):810. doi: 10.1186/s12913-019-4636-6 31699085 PMC6836405

[45] Lee EH, Mancuso JD, Koehlmoos T, Stewart VA, Bennett JW, Olsen C. Quality and Integrated Service Delivery: A Cross-Sectional Study of the Effects of Malaria and Antenatal Service Quality on Malaria Intervention Use in Sub-Saharan Africa. Trop Med Infect Dis. 2022;7(11):363. doi: 10.3390/tropicalmed7110363 36355905 PMC9698472

[46] Negussie A, Girma G. Is the role of Health Extension Workers in the delivery of maternal and child health care services a significant attribute? The case of Dale district, southern Ethiopia. BMC Health Serv Res. 2017;17(1):641. doi: 10.1186/s12913-017-2590-8 28893267 PMC5594610

[47] Pourat N, Lu C, Chen X, Zhou W, Hair B, Bolton J, et al. Trends in access to care among rural patients served at HRSA-funded health centers. J Rural Health. 2022;38(4):970–9. doi: 10.1111/jrh.12626 34617337

[48] Asmamaw G, Minwagaw T, Samuel M, Ayenew W. Availability and readiness of health facilities providing services for other infectious diseases to treat neglected tropical diseases in Ethiopia: implications for service integration in high burden areas. BMC Health Serv Res. 2024;24(1):850. doi: 10.1186/s12913-024-11257-9 39061057 PMC11282672

