# Supplementary material for: Integrated malaria service delivery and its determinants among pregnant women in Ethiopia: Multi level analysis of 2021/22 National Service Provision Assessment Survey
Source: PLoS One. 2026 Apr 15;21(4):e0346831. doi: 10.1371/journal.pone.0346831 (PMC13082725; doi:10.1371/journal.pone.0346831)
Supplement: Attachment — Submitted filename: Response to Reviewers_PloS One_26082025.pdf [file pone.0346831.s002.pdf]

Response to Reviewers' comments

Reviewer #1:

This manuscript investigates the uptake of integrated malaria services and its determinants among pregnant women in Ethiopia. Given the substantial burden of malaria during pregnancy, this is a highly significant topic. To enhance the quality of this manuscript, the following suggestions are put forward:

Response: We sincerely appreciate the reviewer's valuable feedback. We will carefully consider and incorporate the suggestions to further strengthen the quality and clarity of the manuscript.

1. In the "Study Population and Sampling" section, the authors should elaborate on the reasons for excluding 494 facilities within the text.

Response 1:

We thank the reviewer for this important observation. The 494 facilities were excluded because they did not provide antenatal care (ANC) services, and thus no data were collected on ANC or malaria service integration from these facilities. Since our study specifically focuses on malaria service integration within ANC settings, these facilities were not relevant to the analysis and were therefore excluded from the final dataset. We included the revision statement in the method section for clarity.

2. The "Variables and Measurements" section could benefit from reorganization to eliminate redundancy. For example, it is recommended that the paragraph describing the outcome variable be merged with its operational definition.

Response 2:

Thank you for the valuable suggestion. We have merged the similar ideas to operational definition to eliminate the redundancy.

3. In this study, service integration is defined based on three criteria: counseling on insecticide-treated nets (ITNs), the provider's ability to deliver malaria case management in accordance with

national guidelines, and the availability of malaria diagnosis and treatment services at the facility. The authors need to provide more specific details on how these variables were measured using the SPA. Additionally, it would be valuable to explain why the focus was placed on counseling on ITNs rather than the receipt of ITNs or other preventive measures such as IPTp. Furthermore, is there a theoretical foundation for constructing such a combined measurement of service integration?

Response 3:

We are grateful for this insightful comment. The definition of service integration in our study was operationalized using data from the 2021/22 Ethiopia SPA survey at three levels. At the client level, women attending ANC were asked whether they had been counseled on ITN use, which we used to capture malaria-related preventive counseling. At the provider level, information was collected on providers' ability to manage malaria cases in accordance with national guidelines. At the facility level, we used indicators on the availability of malaria diagnostic and treatment services. We combined these three dimensions to construct an index of service integration, drawing on the WHO framework for malaria service integration during pregnancy in malaria-endemic countries.

We focused on counseling on ITNs rather than receipt of ITNs or IPTp because, in Ethiopia, IPTp is not part of the national policy, and ITN distribution to pregnant women is primarily conducted through community campaigns rather than as part of routine ANC services. As such, ITN receipt could not be reliably captured at the facility level in the SPA dataset. Therefore, ITN counseling served as the most valid ANC-based preventive indicator for our integration measure.

4. The authors report on malaria service integration and disparities across regions in Ethiopia. It is important to clarify whether the SPA data can generate representative results for each individual region.

Response 4:
We thank the reviewer for raising this important point. The Ethiopia SPA survey was designed to be nationally representative and stratified by region and facility type. Therefore, the data allow us to make regionally representative estimates of service readiness and integration. We have

clarified this in the revised methods and result (table 1) sections to ensure appropriate interpretation

5. Only a small number of variables mentioned earlier are included in Table 4. Did the authors exclude variables that did not yield significant estimates in the model? Moreover, the difference between Model 1 and the final model should be clearly explained. The authors are advised to report all variables included in both models.

Response 5:
We sincerely appreciate this constructive comment. To evaluate the incremental contribution of different levels of predictors, we compared three multilevel logistic regression models: the null model (intercept only), Model 1 (including only client-level predictors), and the final model (including both client- and facility-level predictors). In Table 4, we reported the results from both Model 1 and the final model side by side. For Model 1, facility-level predictors were not included; hence, these cells were marked with a dash symbol in the table. In the final model, both client- and facility-level predictors were included, and all variables considered in the analysis are presented regardless of their level of statistical significance. This approach ensures transparency and allows readers to see the complete set of predictors included in the modeling process.

Reviewer #2:

Thank you for the opportunity to review this interesting article that examines facility- and client-level factors associated with receipt of integrated MiP services during ANC in Ethiopia. Below are major and minor recommendations to help strengthen this important manuscript.

Response:
We gratefully appreciate the reviewer's insightful suggestions and recommendations. We will consider them to strengthen the importance of the manuscript. For more clarification we provided the responses for each suggestions and feedback point by point.

Major recommendations:

1. Methods: Consistency in use of the language for the model used, i.e. multilevel mixed effects multivariable logistic regression, is needed. What are the fixed versus random effects in the model?

Response 1:

Thank you for the valuable observation. We revised the manuscript to consistently use the term multilevel logistic regression model. In our models, fixed effects included client-level and facility-level predictors, while the random effect was specified at the facility level (Region) to account for clustering of clients within facilities.

2. Did you perform a complete case analysis on your analytic sample? How did you handle missing data?

Response 2:

Thank you for the valuable comment. Variables with minimal missingness (<1%) (e.g., diagnostic availability, supervision) were conservatively recoded, while those with moderate missingness (>1%) (e.g., guideline availability, training) were handled using Multiple Imputation by Chained Equations (MICE) with logistic regression models. The outcome variable was recomputed in each imputed dataset prior to modeling. We have revised the Methods section to reflect this approach.

3. How are health care provider (interviews?) handled in your analyses? Did you aggregate them in some way at the facility level? This is confusing throughout the paper and makes interpretability challenging.

Response 3:

We appreciate this important point. Health care provider data were first linked to the respective facilities and then aggregated to represent facility-level characteristics. For example, provider training and supervision were treated as facility-level predictors in the final models.

4. Is there a reason for listing the Outcome Variable and then the Operational Definition separately? These could be combined as the same thing. It would be helpful to know where each of these variables comes from in the SPA questionnaires. Is there a specific question that corresponds to each? Or are they constructed as composites of questions?

Response 4:

Thank you for the suggestion. We agree and have combined the *Outcome Variable* and *Operational Definition* sections to reduce redundancy. For clarity, we now explicitly state the source of each variable in the SPA questionnaires. For example, ITN counseling was derived

from the client interview dataset, provider capability for malaria diagnosis and treatment was derived from the provider interview dataset, and facility-level diagnostic/treatment availability was obtained from the facility inventory dataset. They have their own questionnaires for each variable. The outcome variable (service integration) was constructed as a composite measure combining these three components, guided by the WHO framework for malaria service integration in pregnancy.

5. Where do data for the malaria regions originate from? SPA or other?

Response 5:

Thank you for raising this. Regional classifications of malaria burden (high, moderate, low) did not originate from SPA but were adopted from the Ethiopian National Malaria Elimination Strategic Plan (2021–2025) and from the national report. We clarified this in operational definition of the Methods.

6. What was your approach to variable selection and inclusion in the adjusted models, beyond model building for multilevel models (i.e. did you select variables a priori based on the literature, based on some cut point, etc? (Table 4)

Response 6:

We appreciate this comment. Our approach to variable selection and inclusion in the adjusted multilevel models was guided by both theory and empirical considerations. Specifically, variables were selected a priori based on the existing literature on malaria in pregnancy (MiP), antenatal care (ANC), and service readiness in low-resource settings. These included key client, provider, and facility characteristics that are recognized determinants of MiP service delivery.

In addition, we performed bivariable analyses, and variables with a liberal significance threshold ($p < 0.20$) were considered for inclusion in the multivariable model. During model building, we applied a stepwise backward elimination strategy guided by the Akaike Information Criterion (AIC) to achieve a parsimonious model. Variables were retained if they improved overall model fit,

This process is described in the Methods and reflected in Table 4.

7. Why was region not included in the models?

Response 7:

Thank you for pointing this out. Region was not included as a fixed-effect predictor because malaria burden region (high, moderate, low) was already incorporated as a categorical predictor, which better aligns with the policy-relevant classification used in Ethiopia. Including both would have introduced collinearity. However, to account for unmeasured variation across regions, we specified region as a random effect in the models.

8. Results/Figures: All figures need some updating to make them interpretable. A map, perhaps of malaria endemicity zones (High, Medium, and Low) and regions would be helpful; better yet if it shows the distribution of facilities. Fig 1 would benefit from a redesign to show how clients cluster within facilities, rather than showing facilities and clients side by side.

Response 8:

We sincerely appreciate the reviewer's constructive suggestion. In line with this feedback, we have redesigned Figure 1**,** the method and result sections to better illustrate the hierarchical structure of the data, where individual clients are nested within facilities. The updated information now emphasizes the clustering of clients within facilities, reflecting the multilevel analytic framework applied in our study. We have also included number of facilities and clients in each region in the result section in Table 1. We believe this update enhances clarity and improves the reader's understanding of the data structure and the rationale for using a multilevel modeling approach.

9. Fig 2 would benefit from reorganizing the key from full to none. The labels as presently listed along the y-axis make this figure difficult to interpret, perhaps due to lack of specificity. Consider using the same language in prose as in the figure to help clarify. Please also clarify whether you are presenting data at the client-level or the facility-level.

Response 9:

Thank you for the feedback. We will reorganize the key in Fig. 2 from "Full" to "None" to improve readability. The y-axis labels will be revised to align with the language used in the text, reflecting the four integration categories: Fully Integrated, Partial, Few, and None. This ensures

consistency between the figure and the manuscript prose. Additionally, we will clarify in the figure legend that the data are presented at the client-level**,** showing the distribution of pregnant women by the integration status of malaria services received during ANC visits.

10. Fig 3 Is the National Average (7.9%) an actual average? I understand it to be the percent of clients who received care at a facility that met the 3-pronged criteria for integrated MiP in ANC, for clients included in your analytic sample. Also, consider relabeling these results - they aren't really a rate. Perhaps frequency would be a better fit.

Response 10:

We appreciate the raised critical points. The national average of 7.9% represents the overall proportion of clients who received care that met the three-pronged criteria for integrated malaria in pregnancy (MiP) services within the study's analytic sample. It is an actual average calculated from the data of all clients included in the analysis. Regarding the labeling, you are correct. "Rate" can be misleading as it often implies a ratio over time or population. Therefore, the figure and related text could be revised proportion.

11. The section on model assessment should either be reduced to a line or two under the results of the model, or put in supplemental files.

Response 11:

We appreciate this suggestion. To improve clarity and focus in the main text, we have streamlined the section on model assessment. In the revised manuscript, only a brief summary line is included under the results to indicate that model assumptions and diagnostics were checked and found satisfactory

Discussion:

12. Consider further exploring in greater detail the effect of cross-sectional data on your results. Were SPA questionnaires administered all at once, or on a rolling basis region-by-region? How does that align / not align with malaria seasonality in Ethiopia? How does timing of client interviews potentially effect integration of MiP in ANC

Response 12:

We thank the reviewer for raising this important point regarding the implications of cross-sectional data. The 2022 Ethiopia SPA survey was conducted all at once rather than on a rolling, region-by-region basis. This timing overlaps with the high malaria transmission season in most Ethiopian regions (August to February), which may have influenced the observed malaria service readiness and integration patterns. We have now added text in the discussion to acknowledge this limitation and highlight that seasonality may have influenced the observed integration patterns.

13. You mention the health extension worker program that includes an element of bed nets and counseling. Please consider going into greater detail about how this could affect your results. If ITNs are not required per national policy as part of MiP services - is this something that is delegated to the CHEWs? Why did you utilize ITN measures but not IPTp, as neither are part of national policy?

Response 13:

We appreciate the comment regarding the Health Extension Worker (HEW) program. In Ethiopia, HEWs are deployed at the community level and their responsibilities include promotion of malaria prevention behaviors, including counseling pregnant women on malaria prevention. While ITN provision is not formally mandated as core malaria in pregnancy (MiP) service in health facility under national ANC guidelines, HEWs routinely carry out this preventive activity as part of the broader community health program.

We focused on ITN counseling in our study because it represents a key, measurable preventive intervention that could be influenced by both facility and community-level service readiness. IPTp was not assessed because, per national policy, IPTp is not part of routine MiP services in Ethiopia.

14. What was the presence of international aid in Ethiopia, by region, at the time of survey? Could that have had a demonstrable impact on your results?

Response 14:

During the 2021–22 SPA data collection period, all regions of Ethiopia received varying levels of international support for malaria programs, including ITN distribution campaigns and facility-

based malaria training supported by partners such as PMI, Global Fund, and ICAP. The presence of international support likely contributed to regional differences in malaria service readiness and integration into ANC. But, the SPA dataset did not systematically capture the geographic allocation of international support during the data collection and we did not consider for the analysis.

15. Consider adding a section (that is supported by your findings) that makes public health recommendations/ addresses implications for national policy and/or programming

Response 15:

Thank you for the valuable suggestions. We will consider adding the public health recommendation and policy implications.

Minor recommendations:

1) needs a thorough copy-edit for typos and grammar

Response: thank you for the comment. We corrected the typos and grammars issues.

2) Double check font type and size in tables and figures (e.g. table 1)

Thank you for the valuable comment. We revised and made similar font size for the tables and figures.

3) Acronyms / abbreviations only need to be spelled out at first use rather than repeatedly throughout the manuscript

Response: We appreciated the comment. We revised to correct the repeated spelled out thought the manuscript.
