# Supplementary material for: Integrated malaria service delivery and its determinants among pregnant women in Ethiopia: Multi level analysis of 2021/22 National Service Provision Assessment Survey
Source: PLoS One. 2026 Apr 15;21(4):e0346831. doi: 10.1371/journal.pone.0346831 (PMC13082725; doi:10.1371/journal.pone.0346831)
Supplement: Attachment — Submitted filename: Point by Point response.pdf [file pone.0346831.s003.pdf]

Point by point responses to the editor and reviewers

Journal Requirements:

1. If the reviewer comments include a recommendation to cite specific previously published works, please review and evaluate these publications to determine whether they are relevant and should be cited. There is no requirement to cite these works unless the editor has indicated otherwise.

   2. Additional Editor Comments:

   There are flaws in methods and results sections especially for multiple statistical models. Please do major revisions as required.

Response to Editor's Comments

We sincerely thank the editor for the thoughtful guidance and for highlighting areas requiring improvement.

1. **Regarding citation of previously published works:**

We have carefully reviewed the references suggested by the reviewers. Where relevant, we have incorporated citations that strengthen the context and interpretation of our findings. For those that were not directly applicable to our study objectives or analytical framework, we have respectfully omitted them, in line with the journal's guidance.

2. **Regarding flaws in the methods and results sections, particularly the use of multiple statistical models:**

   We appreciate this important observation. In response, we have conducted a thorough revision of both the methods and results sections. Specifically:

   - We clarified the rationale for using multilevel logistic regression models, including the hierarchical structure of the data and the nesting of clients within facilities.
   - We refined the description of model specifications, variable selection (based on the WHO MiP framework), and assumptions.
   - We reorganized the presentation of results to improve logical flow and ensure consistency between tables, figures, and narrative text.

We trust that these revisions address the concerns raised and enhance the clarity and rigor of the manuscript. We remain grateful for the opportunity to improve our work and welcome any further feedback.

Review Comments to the Author

Reviewer #1: Thanks the authors for addressing my previous comments. I still have one concern for this manuscript. Although SPA is a nationally representative dataset, it is not safe to assume that the national-level findings also apply to people at the local level. SPA may not be able to generate regionally representative estimates of service readiness and integration. Thus, the authors should be careful about the interpretation of regional-level findings in Figure 3 and the findings on page 13-14.

Response reviewer 1: Thank you for this important and constructive comment. We fully agree that although the SPA is nationally representative, it is not designed to generate fully representative estimates at the individual regional or subnational level. In response, we have revised the manuscript to explicitly acknowledge this limitation and have carefully rephrased the regional-level findings to avoid causal or definitive regional comparisons.

Specifically, we now present regional estimates as descriptive patterns observed within the SPA sample, rather than as population-representative regional prevalence. We emphasize that these findings are intended to illustrate heterogeneity in service integration uptake across settings and to generate hypotheses for future regionally powered studies, rather than to draw firm conclusions about regional performance. This clarification has been added to the Results section (Figure 3 description) ((Line number 361–368) and reinforced in Limitations sections (Line number 519–521).

Reviewer #2: Thank you for your careful attention to initial comments. It appears that some of the figures (e.g. Fig 2) may still require attention based on the prior feedback. Additionally, I would recommend providing more information about study covariates in the methods section.

Additional clarity about use of the outcome variables (integration y/n and the stratified integration variable) is needed. There are two places where the number of levels / models

appears to be incorrectly noted. pg 9: Model performance was compared across the four models using various indices (line 222 in revisions); and pg 7: Variables were categorized into three hierarchical levels (line 161 in revisions).

The authors may also wish to present results stratified by malaria burden area classification or urban/rural status, as a major question remains whether these variables may modify likelihood of receipt of integrated services.

Response to reviewer 2: We thank the reviewer for these helpful and detailed observations.

First, we have revised **Figure 2** to fully address the earlier feedback, improving clarity, labeling, and consistency with the study definitions.

Second, we have expanded the Methods section to provide clearer descriptions of all study covariates, including their conceptual relevance, data source (client, or facility level), and coding approach (line 153-241 in revisions).

Regarding the outcome variables, we have clarified that:

- The primary outcome was a **binary indicator of malaria service integration (yes/no)**; and
- A **stratified integration variable** was used only for descriptive and visualization purposes and not for multivariable modeling.

We also corrected the inconsistencies related to model specification. The final manuscript now clearly states that **three multilevel logistic regression models** were fitted:

1. Null model (intercept-only),
2. Model I (client-level predictors only), and
3. Final model (client- and facility-level predictors combined).

All references to "four models" and unclear hierarchical descriptions have been corrected accordingly in the Methods section (Lines number 379 and 387).

Additional Comment (Reviewer #2)

Comment: The authors may also wish to present results stratified by malaria burden area classification or urban/rural status, as a major question remains whether these variables may modify likelihood of receipt of integrated services.

Response: We appreciate this valuable suggestion. In response, we have incorporated malaria burden area classification and urban–rural location as contextual variables in the analysis and presentation of results. While full stratified multivariable models were beyond the scope of the primary objectives and limited by sample size at subnational levels, we now present descriptive comparisons and discuss the potential modifying role of malaria burden and urban–rural setting in the Discussion section.

We also acknowledge this point as an important direction for future research using datasets with sufficient power for stratified or interaction analyses (Line number 519–521).

Reviewer #3: Thank you to the editor and authors for giving me the opportunity to read this manuscript. I have noted that the authors revised the paper based on other reviewers' comments. However, we believe the manuscript still requires major revisions, particularly regarding the logical flow, operational definitions of outcome variables, and data analysis. My main comments are as follows:

Response to Reviewer #3

We sincerely thank Reviewer #3 for the careful reading of our manuscript and for the constructive comments. We have revised the manuscript extensively in response to these points. Below, we provide a point-by-point response:

1. Title suggests assessment of service uptake, yet uptake is not analyzed.

Response: Thank you for this insightful feedback. In the original version, we used "malaria service uptake" interchangeably with "malaria service integration." We have now revised the manuscript to ensure consistency with the title. Specifically, we have clarified the operational

definition of malaria service uptake and explicitly addressed it in the methods and results sections (see revised operational definitions, Line number 243-246).

2. Line 31 mentions “unborn,” whereas line 59 refers to “newborns.”

Response: Thank you for pointing this out. We have standardized the terminology throughout the manuscript, consistently using “newborns.”

3. Typographical and grammatical errors (e.g., lines 50–52).

Response: We appreciate this observation. The manuscript has been carefully proofread and typographical and grammatical errors have been corrected throughout.

4. Inconsistent use of “integrated malaria services” and “malaria service integration.”

Response: Thank you for highlighting this issue. We have revised the manuscript to consistently use the term “integrated malaria service uptake.” This terminology is based on the WHO framework for integrated malaria services. Ethiopia has adopted this framework for malaria service provision; however, evidence on the level of integrated service uptake among pregnant women remains limited. Our study aims to assess this uptake and its associated factors.

5. Line 109: Unclear whether this is secondary data analysis or cross-sectional study.

Response: Thank you for the clarification request. This study is a secondary data analysis of the Ethiopia SPA Survey 2021/22, which was conducted using a cross-sectional design. We have revised the manuscript to make this clear (line 108-112 in revisions).

6. Figure 1 requires improvement for clarity and logical flow. For example, the arrows from nested clients to facilities appear misplaced.

Response: Thank you for this suggestion. We have revised Figure 1 to improve clarity. The arrows from nested facilities to clients now more clearly indicate that clients receive services from these facilities, reflecting the hierarchical nested structure ((line 141-150 in revisions).

7. Lines 154–155: “Danger sign knowledge” refers only to fever.

Response: Thank you for pointing this out. We have revised the terminology to "Knows fever as a danger sign" and applied this consistently throughout the manuscript ((line 318 in revisions).

8. Lines 164–168: Please explain how the three indicators were derived. Were they from the original survey, or defined by the authors? If self-reported by participants, they may be biased and not reflect provider implementation. For indicators 2 and 3, specify the criteria used for classification.

Response: We appreciate this important request for clarification. The three indicators were derived from existing SPA survey instruments across three datasets: client exit interviews (ITN counseling), provider interviews (capacity to manage malaria cases according to national guidelines), and facility inventories (availability of malaria diagnostics and treatment). These indicators were not self-defined but constructed using predefined SPA variables aligned with WHO service integration guidance. We have expanded the Methods and Operational Definitions sections to explicitly describe the source variables and classification criteria for each indicator (line 153-194 and 242-246 in revisions).

9. In Table 1, font styles are inconsistent. Clarify the meaning of "primary," "secondary," and "higher" education. The proportion of participants who "received ITNs" is lower than those "counseled on ITN use."

Response: Thank you for these observations. Table 1 has been reformatted to ensure consistent font styles. Educational categories are now clearly defined according to the national education system. We also clarified in the text that ITN counseling is provided during ANC visits, whereas ITN receipt often occurs through community-based distribution campaigns, explaining the observed difference.

10. Table 2 and its corresponding text are not informative. They appear to describe sampling methods rather than findings. Did the original study use unequal sampling across facilities?

Response: We appreciate this comment. Table 2 has been revised to more clearly present descriptive findings related to client distribution across facilities and regions, and the accompanying text has been rewritten to emphasize results rather than sampling procedures.

11. Table 3: Define “facility malaria services” and “malaria test lab avai.”

Response: Thank you. We have revised Table 3 to provide clear definitions. “Facility malaria services” refers to the availability of malaria prevention and treatment services, while “malaria test lab availability” refers to the presence of laboratory capacity for malaria testing. We have added clear definitions for these terms in the table footnotes and corresponding methods section.

12. Lines 265–270: Present statistical results for three indicators; clarify lines 276–278.

Response: Thank you. We have revised this section to present the statistical results for the three main indicators more clearly. Lines 276–278 have been rewritten for clarity in revised method section (line number 163-202 and in result section (line 343-349).

13. Table 4 should be expanded. The title is vague. Include cross-tabulated data and corresponding simple logistics regression results (COR etc.) to substantiate the findings. If multiple models were tested, include all results as supplementary material. Variables such as “supervision received” and “facility malaria training,” which appear here but not in prior tables, must be defined and linked to earlier sections.

Response: Thank you for this important and constructive comment. We have revised Table 4 extensively to improve clarity, transparency, and interpretability. The table has been retitled to explicitly indicate that it presents results from multilevel mixed-effects logistic regression models examining factors associated with integrated malaria service uptake.

All variables included in the multivariable models are now clearly defined in the Methods and Operational Definitions sections and are consistently referenced throughout the manuscript (line 215-241 in revisions).

To enhance transparency of model building, results from all tested models (null model, client-level model, and full model) are now presented, with the main text focusing on the final adjusted model (line number 375-382 in revision). Variables such as *supervision received* and *facility malaria training* have been explicitly defined and linked to earlier tables describing provider- and facility-level characteristics (line 234-239 in revisions).

14. Discussion section is lengthy and repetitive.

Response: Thank you for this important observation. We have shortened and streamlined the Discussion to reduce repetition, particularly regarding integration of malaria services. We have expanded the section on associated factors and included references to more relevant studies to strengthen the discussion.

Overall: We greatly appreciate Reviewers' constructive comments. These revisions have substantially improved the clarity, consistency, and rigor of the manuscript.
