# Supplementary material for: Integrated malaria service delivery and its determinants among pregnant women in Ethiopia: Multi level analysis of 2021/22 National Service Provision Assessment Survey
Source: PLoS One. 2026 Apr 15;21(4):e0346831. doi: 10.1371/journal.pone.0346831 (PMC13082725; doi:10.1371/journal.pone.0346831)
Supplement: Attachment — Submitted filename: Point by point responses.docx [file pone.0346831.s004.docx]

**Response to Reviewer #2**

**Comment:** Needs a typographical / grammar review by authors. There are many instances where additions / revisions now read with grammatical errors. For example, what is "few integration"?

**Response:** Thank you for this important observation. We acknowledge that some revisions introduced grammatical inconsistencies and unclear terminology. The manuscript has now undergone a thorough language and typographical review to improve clarity and readability. Terms such as *“few integration”* have been revised to *“limited integration”* throughout the manuscript to ensure consistency and correct interpretation. All sections have been carefully edited for grammar, terminology, and sentence structure.

**Response to Reviewer #3**

Thank you for your continued careful review and constructive feedback. We sincerely appreciate the opportunity to further improve the manuscript.

**Comment 1:** The outcome variable was dichotomized as 1 and 0. Please clarify how partially integrated or few integrated categories were handled.

**Response:** Thank you for this important request for clarification. The integration variable was originally constructed as a four-level composite indicator:

- **Full integration** – all three components present
- **Partial integration** – two components present
- **Limited integration** (few integration) – one component present
- **No integration** – none present

For regression analysis, this composite measure was dichotomized to reflect the presence of fully integrated malaria service uptake:

- 1 = Fully integrated (all three components available and delivered)
- 0 = Not fully integrated (partial, limited, or no integration)

Thus, the categories “partial” and “limited” were retained for descriptive analysis but combined under the reference group in the multilevel regression models. We have clarified this in the Methods and Operational Definitions sections.

**Comment 2:** The outcomes appear to measure facility performance rather than service uptake by participants.

**Response:** We appreciate this insightful distinction. Our outcome reflects integrated malaria service uptake at the point of care, defined as the receipt of ANC in a setting where malaria prevention counseling, provider capability, and facility readiness were simultaneously available during the visit.

While provider capacity and facility readiness are supply-side indicators, their co-occurrence with client-level counseling reflects the actual opportunity for pregnant women to receive integrated malaria services during **ANC**. Therefore, the outcome captures functional service uptake, rather than mere attendance.

To avoid ambiguity, we have revised the manuscript to clarify that the study assesses:

*“uptake of integrated malaria services within ANC encounters”* rather than individual behavioral uptake alone.

**Comment 3: VIF vs p-value selection**

**Comment:** Line 296 mentions the use of VIF, while Line 333 refers to excluding variables with p < 0.2. How were these criteria applied when they potentially conflict with each other?

**Response:** Thank you for highlighting this. The criteria were applied sequentially:

- Variables were first selected for multivariable modeling using a p < 0.2 threshold in bivariate screening.
- Multicollinearity among selected variables was then assessed using Variance Inflation Factor (VIF) and all variables were VIF< 5 were included in the analysis.

Thus, statistical relevance (p < 0.2) guided inclusion, while VIF ensured independence among predictors.

**Response 4:** Thank you for noting these remaining issues. The following revisions have now been made:

- **Figure 1** redesigned to clearly reflect client clustering within facilities.
- **Table 1** font and formatting standardized.
- **Table 2** revised to better reflect analytical relevance and contextualized in text.
- **Table 3** variable names clarified and defined.
- **Table 4** expanded and retitled; variables are now defined earlier and model structure clearly explained.

Table 4 presents the results of the multilevel logistic regression analysis examining factors associated with integrated malaria service uptake.

Cross-tabulated results and crude odds ratios (COR) were not included in this table because the analysis was conducted using a hierarchical multilevel modeling framework, which simultaneously accounts for client-level and facility-level clustering effects. In this context, simple cross-tabulations do not appropriately reflect the nested data structure and may produce biased or misleading estimates.

Instead, Model I present the client-level adjusted estimates, while the Final Model presents estimates adjusted for both client- and facility-level factors. This approach allows for appropriate estimation of associations while accounting for intra-facility correlation.

- The **Discussion** section has been streamlined to improve logical flow and reduce repetition.

We sincerely appreciate your continued guidance, which has helped strengthen the clarity and methodological transparency of the manuscript.
