# Supplementary material for: Integrated malaria service delivery and its determinants among pregnant women in Ethiopia: Multi level analysis of 2021/22 National Service Provision Assessment Survey
Source: PLoS One. 2026 Apr 15;21(4):e0346831. doi: 10.1371/journal.pone.0346831 (PMC13082725; doi:10.1371/journal.pone.0346831)
Supplement: Attachment — Submitted filename: Point bypoint responses.docx [file pone.0346831.s005.docx]

**Point by point responses to editor and reviewers**

Review Comments to the Author

Reviewer #2: 1) It does not appear a thorough grammar review was conducted as was recommended previously. For example, (and this is one of many instances): "The finding is consistent with pervious study that the association of integrated antenatal and malaria service quality scores with ITN access [44]." I cannot tell what the authors are trying to state about reference 44 in relation to their findings based on the way this sentence currently reads.

**Response to Reviewer #2, Comment 1:**

Thank you for this important observation. We agree that the sentence was grammatically unclear and did not adequately convey the intended meaning. In response, we carefully revised the wording throughout the manuscript to improve grammar, clarity, and readability. The cited sentence has been corrected to clearly state the relationship between our finding and the previous study. It now reads: “This finding is consistent with previous evidence showing that higher quality integrated antenatal and malaria services are positively associated with improved ITN access [45].” We also conducted an additional language review across the manuscript to address similar issues and improve overall clarity.

2) I agree with reviewer two that the terminology 'service uptake' is challenging here because the authors are constructing their outcome based on structural and process factors. This seems much more in line with Service Availability and Readiness, not whether women necessarily used the services (e.g. for those who received an ITN, did they employ use of an ITN more often following receipt counseling/education). Indeed, the operational definition is: "Receipt of coordinated malaria-related services during ANC, defined by the concurrent presence of malaria prevention counseling, provider capacity for malaria case management, and facility readiness for malaria diagnosis and treatment. The authors should consider reframing their terminology to be a more accurate reflection of what is actually being measured. How are these constructs used and defined in the published literature?

**Response to Reviewer #2, Comment 2:**

Thank you for this important conceptual clarification. We agree that the term *“service uptake”* may imply individual behavioral utilization, whereas our outcome variable reflects the **delivery of integrated malaria services during ANC encounters**, based on structural and process indicators derived from the SPA survey.

In response, we have revised the terminology throughout the manuscript to **“integrated malaria service delivery during ANC.”** This terminology more accurately reflects the constructs being measured, which combine:

- Client-reported malaria prevention counseling (process indicator),
- Provider capacity to manage malaria cases (provider-level readiness), and
- Facility readiness for malaria diagnosis and treatment (structural readiness).

These indicators align conceptually with **health service integration and service readiness frameworks**, including the WHO **Service Availability and Readiness Assessment (SARA)** and the **malaria in pregnancy (MiP) service delivery framework**.

The Introduction, Methods, Operational Definitions, and Results sections have been revised accordingly to ensure conceptual consistency between the study objective, outcome definition, and interpretation of findings.

Reviewer #3: Upon my quick reassessment of the revised manuscript, I find that the revisions are superficial and do not adequately address the concerns raised in the previous round of review. In many instances, no substantive changes have been made. Therefore, my comments remain the same as before.

**Response to reviewer #3 comments:**
Thank you for your careful reassessment and for emphasizing the need for more substantive revisions. We sincerely acknowledge that our previous revision did not fully address the conceptual concerns raised, particularly regarding the definition and interpretation of the outcome variable.

In this revision, we have made substantial and targeted changes to address your concerns. Most importantly, we have **reframed the primary outcome and revised the manuscript accordingly** to ensure conceptual clarity and alignment with the data source and analytical approach.

We fully agree with this important observation. Upon reconsideration, we recognize that the original terminology “service uptake” did not accurately reflect the nature of the outcome variable, which is derived from a combination of **client-reported counseling (process indicator), provider capacity, and facility readiness (structural indicators)**.

To address this, we have **reframed the outcome throughout the manuscript** as:

**“Integrated malaria service delivery during antenatal care (ANC)”**

This revised terminology more accurately reflects that the study measures the **concurrent delivery of key malaria service components during ANC encounters**, rather than individual behavioral uptake by clients.

We have also clarified that the outcome represents a **composite measure of service integration**, aligned with health system frameworks such as the WHO Service Availability and Readiness Assessment (SARA), rather than a direct measure of utilization or adherence.

We believe these revisions substantially address the core conceptual concern raised and improve the validity, clarity, and interpretability of the study. We are grateful for your critical feedback, which has significantly strengthened the manuscript.
